# C-terminal glycosylation of type IX secretion system cargo proteins in *Prevotella intermedia* with both short and long secretion signals

**DOI:** 10.1098/rsob.240335

**Published:** 2025-03-26

**Authors:** Xi Ye, Nabil bin Rustam, Dhana Gorasia, Eric Reynolds, Debnath Ghosal, Paul Veith

**Affiliations:** ^1^ Department of Biochemistry and Pharmacology, The University of Melbourne, Melbourne, Victoria, Australia; ^2^ Melbourne Dental School, The University of Melbourne, Melbourne, Victoria, Australia; ^3^ ARC Centre for Cryo-electron Microscopy of Membrane Proteins, The University of Melbourne, Melbourne, Victoria, Australia

**Keywords:** *Prevotella intermedia*, alaninamide, glycosylation, protein secretion, secretion signal, mass spectrometry

## Abstract

*Prevotella intermedia* is a Gram-negative bacterium that is associated with periodontitis and acute necrotizing ulcerative gingivitis. *P. intermedia* utilizes the type IX secretion system (T9SS) to secrete and anchor virulence factors to the cell surface, presumably via C-terminal glycosylation. The identity of the linking sugar and the sites of modification on the cargo are unknown. Here, we employed hidden Markov models to predict cargo proteins in *P. intermedia* and conducted LC-MS/MS analyses of partially deglycosylated fractions to characterize the C-terminal glycosylation. A total of 80 cargo proteins were predicted based on the presence of a T9SS C-terminal domain (CTD) signal, and these were divided into 48 short CTDs and 32 long CTDs. Cleavage sites for five short and four long CTDs were experimentally determined, and glycosylation was observed at the mature C-terminus of six cargo. Two glycans were identified of delta masses 419.198 and 433.185 Da, corresponding to novel C-terminal amide linkages to N-alanyl dHex-HexNAc and N-alanyl (Me-dHex)-HexNAc, respectively. This indicated that both short and long CTDs supported cleavage and glycosylation. AlphaFold multimer modelling predicted that both kinds of CTDs could bind to the PorV shuttle protein in the same manner, with the conserved CTD motifs interacting with the same sites in PorV.

**Figure:**
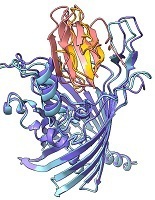

## 1. Introduction

*Prevotella intermedia* is a Gram-negative, black-pigmented, non-motile, rod-shaped, obligate anaerobic bacterium that belongs to the *Bacteroidota* phylum. It is routinely found in subgingival dental plaque and is associated with periodontitis, a chronic inflammatory disease that leads to the destruction of the tooth’s supporting tissues including the alveolar bone [1]. Studies have indicated a positive correlation between the presence of *P. intermedia* in subgingival plaque and clinical measurements of chronic periodontitis, including increased pocket depth [2], clinical attachment loss [3] and bleeding upon probing [4]. Additionally, *P. intermedia* has been identified in extraoral sites such as NOMA (cancrum oris) lesions [5] and bacterial tracheitis in children [6]. Notably, it is the only periodontal pathogen known to induce severe bacteremic pneumococcal pneumonia, accompanied by enhanced pneumococcal adhesion to lower airway cells [7]. As a pathogen, several adhesins (AdpC/F) [8,9], the cysteine proteinase interpain A [10] and LPS [11] have been identified as virulence factors in *P. intermedia*, yet the mechanisms underlying the bacterium’s virulence remain poorly understood.

The type IX secretion system (T9SS) is the ninth protein secretion system in bacteria that is confined to the *Fibrobacteres-Chlorobi-Bacteroidota* superphylum [12]. While the T9SS was recently studied in *P. intermedia* for the first time [13], it has been well-studied in *Porphyromonas gingivalis* and *Flavobacterium johnsoniae* [14–17]*. Additionally,* a recent bioinformatic prediction identified the T9SS in 402 out of 693 examined *Bacteriodota* strains [18]. Briefly, T9SS substrates (cargo proteins/C-terminal domain [CTD] proteins) exhibit characteristic N-terminal signal peptides to direct transport across the inner membrane (cytoplasmic membrane) via the Sec system into the periplasm [19]. They are subsequently folded and targeted to the Sov (SprA) translocon, a large 36-stranded β-barrel outer membrane protein (OMP) with a periplasmic entrance and a lateral gate through the barrel wall on the extracellular side of the outer membrane (OM). In its secretion-ready state, the smaller 14-stranded β-barrel OMP, PorV is bound to the lateral gate where it collects the cargo proteins from the lumen of the translocon by binding to their conserved CTD signals [20,21]. PorV then shuttles the cargo protein to the cell surface attachment complex, which includes the PorU sortase [22–25], and a polysaccharide-binding protein, PorZ [26,27]. The PorU sortase, which contains a gingipain-like protease domain, cleaves the conserved CTD signal of the exported T9SS cargo. Previous bioinformatic studies demonstrated that the CTD cleavage sites, as well as three motifs in the cleaved CTD, are conserved across several *Bacteroidota* species [28], suggesting a conserved process of CTD recognition and cleavage. Upon CTD cleavage of T9SS cargo proteins, the PorU sortase covalently conjugates the cargo’s new C-terminus to a lipopolysaccharide anchor [24]. This attachment ultimately leads to the formation of a ‘virulence coat’ around the cell [29].

Through the process of OM budding, virulence factors that attach to the bacterial surface are packaged into the outer membrane vesicles (OMVs), which are actively released to exert their virulence, including synergistic growth promotion of other microorganisms, adhesion and penetration into the host epithelium, as well as host immunomodulation [17,30,31]. Since the OMVs of *P. intermedia* and other T9SS-positive bacteria are enriched with T9SS cargo, the OMV release in these organisms can be considered an extension of the T9SS [32–34].

LPS is a major component of the OM of Gram-negative bacteria that maintains its structural integrity and mediates interactions with the external environment [35]. It consists of a hydrophobic lipid A that constitutes the outer leaflet of the OM, a core oligosaccharide and an *O*-polysaccharide composed of repeating sugar units. The ‘linking sugar’ that links LPS to the new C-terminus of T9SS cargo proteins has been determined to be 2-N-seryl, 3-N-acetylglucuronamide in *P. gingivalis* and 2-N-glycyl, 3-N-acetylmannuronic acid in *T. forsythia* [36]. These linking sugars are synthesized through the Wbp/Vim pathway where the Wbp enzymes produce a 2N,3N-diacetylated glucuronic acid and the Vim enzymes deacetylate at the 2N position which is subsequently N-acetylated with an amino acid, either serine for *P. gingivalis* or glycine for *T. forsythia* [36]. The form of *P. gingivalis* LPS containing the linking sugar is called A-LPS, and recent data suggest that A-LPS may actually be a form of *O*-LPS with the linking sugar located on a side or terminal branch of the *O*-polysaccharide to allow the linkage to cargo proteins [36,37].

In addition to C-terminal cargo glycosylation with LPS, *P. gingivalis* and *T. forsythia* also have the Bacteroidota general *O*-glycosylation system which glycosylates hundreds of exported proteins at the D(S/T)X motif [38,39]. Our recent study demonstrated the *O*-glycosylation system in *P. intermedia* for the first time [34]. Forty-seven distinct T9SS cargo proteins were identified, 14 of which, including key virulence factors such as adhesins, internalins, peptidases, hemin-binding protein and leucine-rich repeat (LRR) domain-containing protein, were revealed to be *O*-glycosylated. Among the 19 T9SS protein components identified in *P. intermedia* so far, nine were found to be O-glycosylated, including PorE/F/K/M/N/W/Y/U and Sov [34]. Mutants deficient in PorK or PorT demonstrated that the T9SS is essential for black pigmentation, hemagglutination, biofilm formation and functioning of cell surface virulence factors in *P. intermedia* [13]. Interestingly, *P. intermedia* is the only species observed to have short CTDs with many containing only 50−60 residues compared to more than 70 residues in other species examined [28]. In the same study, more than 10 cargo proteins of *P. intermedia* were substantially elevated in molecular weight, consistent with LPS modification; however, the modification sites and attached sugars are yet to be determined. The structure of the lipid A portion of *P. intermedia* LPS is known to be monophosphorylated and penta-acylated [11,40]; however, the structure of the core oligosaccharide and polysaccharide portions is unknown.

In this study, by analysing partially deglycosylated fractions of *P. intermedia*, we identify the CTD cleavage sites and the linking sugars that anchor the T9SS cargo proteins on the cell surface. We show that cargo proteins with either short or long CTDs can be cleaved and modified, and both are predicted to be shuttled via PorV.

## 2. Material and methods

### 2.1. Growth of *P. intermedia*

*P. intermedia* ATCC 25611 was grown in 25 g l^−1^ brain-heart infusion broth and 30 g l^−1^ tryptic soy broth, supplemented with 5 µg ml^−1^ hemin, 1 µg ml^−1^ vitamin K and 0.5 g l^−1^ cysteine under anaerobic conditions (80% N_2_, 10% CO_2_ and 10% H_2_) at 37°C for 24 hours.

### 2.2. Cell fractionation and isolation of OMVs

*P. intermedia* cells were harvested and fractionated following the protocol employed in a previous study [34]. As CTD proteins are transported to the cell surface via the T9SS, the membrane and OMV fractions were the focus for analysis.

### 2.3. Partial deglycosylation

Portions of membrane fraction, OMVs and TCA-precipitated soluble fraction were resuspended in 50% acetonitrile−0.1% aqueous trifluoroacetic acid (TFA), transferred to reaction vials and freeze-dried thoroughly overnight. Deglycosylation was performed following the protocol provided by the manufacturer of the ProZyme/Glyko Glycofree chemical deglycosylation kit (GKK-500) as previously described [34].

### 2.4. SDS-PAGE and in-gel digestion

Deglycosylated samples were dissolved in 1 × NuPAGE LDS sample buffer and 50 mM dithiothreitol to denature proteins. After sonication and heating, all samples were separated by reducing SDS-PAGE and fractionated into 12 gel segments, respectively. The segments were digested with trypsin in the gel as described previously [24] and extracted once with 0.1% aqueous TFA and once with 30% acetonitrile−0.1% aqueous TFA, both for 15 minutes, in an ultrasonication bath. Extracts were combined, evaporated in a vacuum centrifuge and dissolved in 2% acetonitrile−0.1% aqueous TFA for MS analysis.

### 2.5. Identification of CTD cleavage sites

Culture fluid from *P. intermedia* was centrifuged at 170 000 g for 3 hours to pellet the vesicles. The supernatant was collected and concentrated using a 1 kDa centrifugal filter. The concentrate was loaded onto an SDS-PAGE gel and separated using MES running buffer. The gel was stained with Coomassie, and the region below 10 kDa was excised into four segments for in-gel digestion with trypsin.

### 2.6. Mass spectrometry

LC-MS/MS experiments were conducted on a Dionex Ultimate 3000 UHPLC interfaced with an Orbitrap Fusion Lumos Tribrid mass spectrometer (Thermo Fisher Scientific) as previously described [39], with the following modifications. Membrane and OMV samples were eluted using a 60 minute gradient of approximately 2–32% ACN. A FAIMS method alternating between −25 V and −45 V was utilized, and only HCD spectra were collected. For the acquisition of CID spectra, an inclusion mass list of the previously identified glycopeptide ions was used to trigger the CID scans in separate experiments. For the identification of CTD cleavage sites from culture fluid samples, a standard Orbitrap proteomics method (not FAIMS) was employed.

### 2.7. Peptide identification

Proteins and peptides were identified by searching against the *P. intermedia* ATCC 25611 sequence database of 2156 protein sequences downloaded from Uniprot Proteomes (Proteome ID = UP000187195). All searches were performed using trypsin and other parameters were as follows. Maximum missed cleavages = 2, peptide mass tolerance = 10  ppm, fragment mass tolerance = 0.04  Da, fixed modification = cysteine carbamidomethyl and variable modifications = methionine oxidation. Initial searches were conducted using Byonic v4.6 (Protein Metrics, US) with a semi-specific C-ragged search in wildcard mode, allowing any modification mass up to 1000 Da to be identified. Once the modifications were known, the same data were also searched using Mascot v2.8.2 (Matrix Science, UK) using semi-trypsin specificity and with the additional custom variable modifications C_17_H_27_N_3_O_8_ (401.1798 Da), C_17_H_29_N_3_O_9_ (419.1904 Da) and C_18_H_31_N_3_O_9_ (433.2060 Da) applied to any C-terminus. The data were analysed in the following manner. The result files of the Byonic searches were exported to Excel via Byonic Viewer v4.6. The data were filtered to only include modified C-ragged peptide sequences located within the last 100 C-terminal amino acids of the CTD proteins. The data were then manually inspected to find common Δmass values. The corresponding spectra were then checked to ensure correct assignment of monoisotopic peak, charge state, strong series of b-ions and once known, the presence of the HexNAc ion at m/z 204.086. For CTD cleavage site analysis, Mascot searches were conducted as above with semi-trypsin specificity but without the glycan modifications.

### 2.8. Bioinformatics

The initial list of T9SS cargo (CTD proteins) for *P. intermedia* ATCC 25611 was generated from the list of CTD proteins predicted for a different strain (*P. intermedia* 17) [28]. This was achieved using BLAST. From this list, 23 protein sequences with long CTDs and 22 protein sequences with short CTDs from *P. intermedia* ATCC 25611 were aligned using Clustal-Omega (v.1.2.4) software. Multiple sequence alignments of long and short CTDs were treated as inputs for the generation of hidden Markov model (HMM) profiles [41] which were then searched against the *P. intermedia* ATCC 25611 protein database for CTD protein prediction using HMMER (v.3.3.2). An inclusion threshold of 1e−1 was used. As the size of the *P. intermedia* proteome is small, the chance of finding random matches is low. Thus, a more relaxed threshold was used than the default 1e−5. Moreover, hits were only accepted if they extended to the C-terminus.

Conserved domains in predicted CTD proteins were found using NCBI Batch CD-Search. The conserved pectin lyase domains were detected using InterProScan. The hits were extended by the detection of homologs using BLAST. Signal peptides and their cleavage sites were predicted using SignalP 6.0 [42]. Transmembrane domains were predicted using TMHMM 2.0 [43]. Motif logos were generated using WebLogo v2.8.2 (https://weblogo.berkeley.edu/logo.cgi) [44].

AlphaFold models of cargo proteins were downloaded from the AlphaFold Protein Structure database (https://alphafold.ebi.ac.uk) and AlphaFold 3 Multimer predictions for the PorV-cargo complexes were achieved using the AlphaFold server (https://alphafoldserver.com) using default parameters. Protein models were visualized and aligned using UCSF ChimeraX v1.8 [45]

### 2.9. Glycan nomenclature

The nomenclature employed for depicting and abbreviating sugars was adopted from the Symbol Nomenclature for Glycans (SNFG) (https://www.ncbi.nlm.nih.gov/glycans/snfg.html) [46].

## 3. Results

### 3.1. Prediction of T9SS cargo (CTD proteins)

Using the 45 CTD proteins predicted in our previous study [28], multiple sequence alignments were assembled for the creation of HMMs. The initial alignments showed that the CTD proteins could be divided into two sub-groups based on length. The first group exhibited 22 short CTDs ranging from 40 to 51 amino acid residues (aa) from predicted cleavage site (see below) to C-terminus while the second group contained 23 long CTDs that ranged from 69 to 82 aa (electronic supplementary material, figure S1 and S2). HMMs were created for both groups and used to search against a *P. intermedia* sequence database. In total, 80 CTD proteins were predicted including the seed proteins (electronic supplementary material, table S1). Of these, 61 and 44 were identified using the short and long HMMs, respectively, with 25 overlapping. The predicted proteins were validated by (i) confirming that the hit sequences were located at the C-termini; (ii) confirming the presence of an N-terminal signal peptide; and (iii) confirming the presence of a predicted CTD-like domain in the AlphaFold-predicted structures (electronic supplementary material, table S1). The 80 predicted T9SS cargo proteins were also analysed for conserved domains. The domain architectures of cargo with known functional domains or domains that were commonly identified among the 80 sequences were plotted (electronic supplementary material, figure S3 and S4A). Common architectures included proteins dominated by LRR regions, proteases and putative adhesins with fibronectin type 3 and choice-of-anchor J domains (electronic supplementary material, figure S3). Proteins composed of 1−3 copies of the pectin lyase domain were also common (electronic supplementary material, figure S4A). The pectin/pectate lyase fold is a β-helix composed of three parallel β-strands per turn, with a roughly triangular cross-section (electronic supplementary material, figure S4B and S4C). The LRR domains were also β-helical, but each ~24 residue repeat was predicted to form an irregular cloud-shaped cross-section incorporating just one β-strand per turn (electronic supplementary material, figure S4D and S4E). A highly conserved AF motif was located at the base of the most prominent invagination (electronic supplementary material, figure S4E and S4F). The DUF285 domain as exemplified in A0A1P8JEP3 formed a curved structure composed of a repeating unit of similar cloud-like cross-section but without any β-strands (electronic supplementary material, figure S4G and S4H). The conservation within the 26-residue repeat was very high and included an MF motif at position 3−4 and a further F at position 18 (electronic supplementary material, figure S4I). The F sidechains in both these repeats stack together providing stabilization for the fold. No obvious correspondence between CTD length and function was observed.

### 3.2. Determination of the masses of linking glycans

Following on from our previous studies where we observed highly modified CTD proteins in *P. intermedia* [28] and developed deglycosylation methods for characterizing similarly modified CTD proteins in *P. gingivalis* and *T. forsythia* [36], membrane and OMV fractions of *P. intermedia* were partially deglycosylated and subjected to SDS‐PAGE, excision into 12 gel segments, in‐gel digestion with trypsin, and LC‐MS/MS analysis. Potential C-terminal glycopeptides were detected through a semi-specific C-ragged search in Byonic wildcard mode, which allowed the identification of peptides from CTD proteins modified with any delta mass (Δmass) value. The location of these peptides within the protein sequences was then inspected to determine their proximity to the CTDs of T9SS cargo. Subsequently, these C-terminal peptides, particularly the frequently observed ones with non-tryptic cleavages, were selected to search for the most frequent Δmass values. Two promising C-terminal peptides were found with common Δmass values of 418/419 and 433  Da (electronic supplementary material, table S2) and checking the MS2 spectra for these peptides confirmed they were glycosylated at the C-terminus (see below). After checking the accurate assignment and mass of the monoisotopic peaks in the MS1-level spectra, the Δmass values of 418 Da were found to be 419 Da. Furthermore, the accurate masses were calculated to be 419.198 and 433.185 Da, respectively. By incorporating these two masses as variable modifications in the Mascot search, C-terminal peptides that were putatively modified by these linking glycans were established. Six unique peptides from six different CTD proteins were identified and validated by manual checking of the MS2 spectra (table 1). In the process of checking the data, longer C-terminal modifications for example those utilizing additional sugars could not be found, however one shorter modification of Δmass 401 was observed (table 1).

**Table 1.** Identification of modified C-terminal peptides by Mascot searches.

accession	peptide sequence	score	E-value	mass^EXP^ ^a^	Δmass^b^
A0A1P8JBQ3	SHAYNSAPVLYLGFDVAN	80.9	2.6E−08	2338.119	401.187
A0A1P8JBQ3	SHAYNSAPVLYLGFDVAN	98.7	5.5E−10	2356.127	419.195
A0A1P8JBQ3	SHAYNSAPVLYLGFDVAN	80.0	3.2E−08	2370.151	433.219
A0A1P8JF82	AYIIGHDGLDKLEILFNGDFVT	66.2	6.3E−07	2850.429	401.176
A0A1P8JF82	AYIIGHDGLDKLEILFNGDFVT	90.3	3.5E−09	2868.446	419.193
A0A1P8JF82	AYIIGHDGLDKLEILFNGDFVT	86.0	8.6E−09	2882.457	433.204
A0A1P8JDA6	KYSSDPLTTEVVYA	26.6	0.0032	1990.960	419.189
A0A1P8JDA6	KYSSDPLTTEVVYA	47.3	3.7E−05	2004.976	433.204
A0A1P8JFA6	GAEDLHPTAD	17.4	0.024	1457.650	433.204
A0A1P8JG72	GVVSNKPLTVVQGDITA	31.3	0.0012	2130.139	433.203
A0A1P8JC41	LGNSAIPM_ox_T…M_ox_VEVTTTA	18.5	0.018	3083.484	419.185

^a^ Experimental neutral mass of the modified peptide.

^b^ Δmass = mass (experimental) − calculated mass of peptide only.

### 3.3. Identification of the glycan sequences based on MS/MS analyses

**Figure 1.**
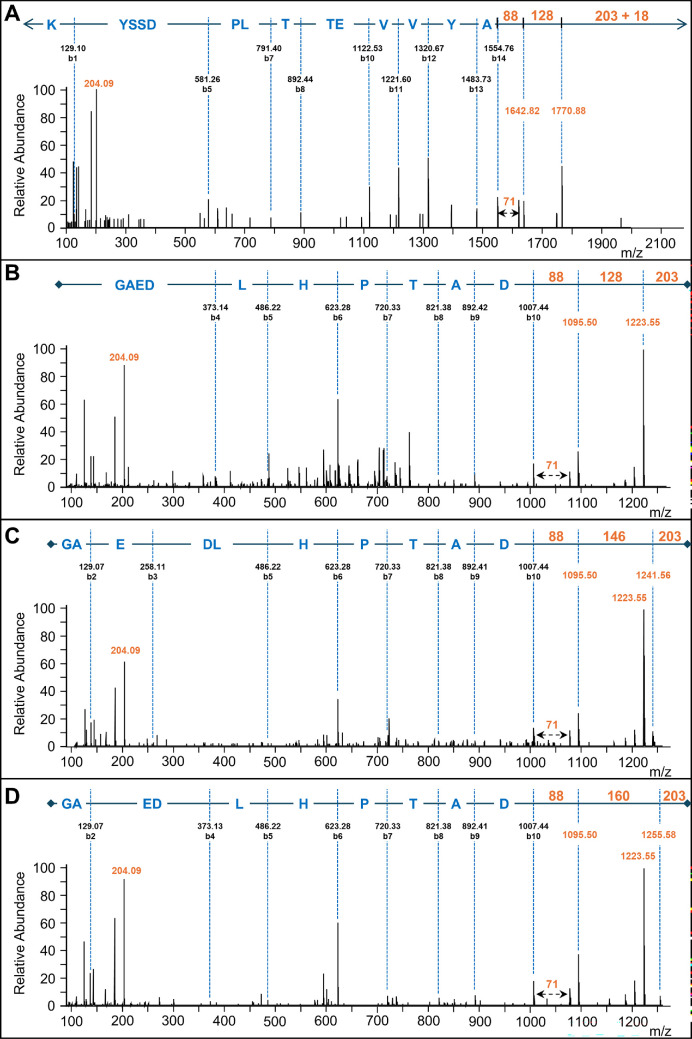
HCD spectra of modified C-terminal peptides. Modified CTD proteins were deglycosylated with TFMS, in-gel digested with trypsin and analysed by LC-MS/MS. (A) Showing the peptide KYSSDPLTTEVVYA of the CTD protein A0A1P8JDA6, which was modified by the 419 Da linker. The precursor m/z was 996.49 (2^+^). (B–D) Showing the peptide GAEDLHPTAD from the CTD protein A0A1P8JFA6 modified with Δmass values of 401, 419 and 433 Da from precursor m/z values of 713.82 (2^+^), 722.83 (2^+^) 729.83 (2^+^), respectively. For all spectra, the masses of the matching b-ions are shown revealing the peptide sequence. The masses of the linker components appended to the C-terminus of each peptide are shown in orange. All labelled ions are singly charged. The labelled 204.09 m/z peak corresponds to the HexNAc oxonium ion. The mass differences of 88, 146, 160 and 203 correspond to alaninamide, dHex, Me-dHex and HexNAc, respectively.

To determine the sequences of the two linking glycans conjugated to the new C-terminus of CTD proteins, the corresponding HCD and CID spectra were analysed. Initially, the low-mass region of HCD spectra was manually inspected to identify any recurring peak that could potentially correspond to a sugar moiety. A robust peak at 204.09 *m/z* was present in the spectra of all modified peptides (figure 1), which matches to a HexNAc oxonium ion. The HCD spectra of four of the modified peptides revealed a series of N-terminal fragment ions (b-ions) corresponding to each amino acid residue of the peptide, followed by the linking sugar residues (figure 1, electronic supplementary material, figure S5). For the peptide KYSSDPLTTEVVYA+419 (figure 1A), after the C-terminal amino acid (Ala), the b-ions continued with peaks of+88 and a further+128 Da leaving 221 Da to reach the mass of the precursor ion. For the peptide GAEDLHPTAD, three variants were found with Δmass values of 401, 419 and 433 (figure 1B–D). The first of these, Δmass = 401 (figure 1B), shows consecutive peaks at+88 and+128 Da leaving 203 Da (HexNAc) to reach the precursor ion. For Δmass 419 and 433, the same peaks at 1095 (+88) and 1223 Da (+128) were observed; however, additional peaks at 1241 (figure 1C) and 1255 Da (figure 1D) were seen to account for the additional mass. Keeping the final mass difference of 203 Da constant, corresponding to HexNAc, the middle portion of the modification has a mass of 128, 146 or 160 Da, respectively.

Based on the accurate mass, the first fragment (88.064 Da) matched only to the molecular formula C_3_H_8_N_2_O, which matches to alaninamide among others. The presence of an additional peak at+71.037 Da in all spectra (figure 1), corresponding to an Ala residue, further supported that the first fragment was an alaninamide. For the major Δmass forms of+419 and+433, the middle portions of 146.057 and 160.072 Da closely matched a deoxyhexose residue (dHex) and an O-methylated dHex residue, respectively. The more dominant+128 Da peak can be explained by the loss of water (H_2_O, 18 Da) from the dHex or methanol (CH_3_OH, 32 Da) from the Me-dHex. At this stage, the 419 and 433 Da linking glycans were postulated to be alaninamide-dHex-HexNAc and alaninamide-(Me-dHex)-HexNAc.

**Figure 2.**
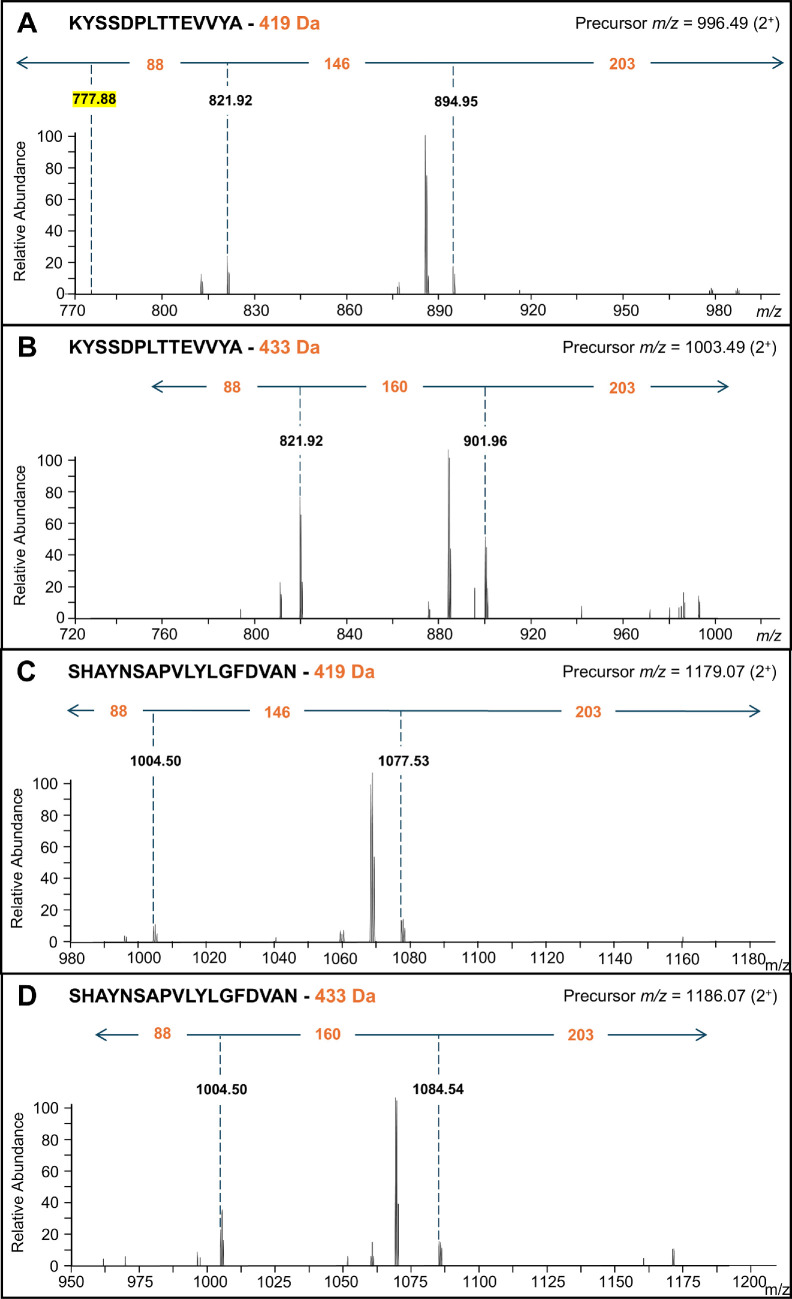
CID spectra establishing the components of 419 and 433 Da linking glycans. (A and B) were obtained from the peptide KYSSDPLTTEVVYA of the CTD protein A0A1P8JDA6, which were modified with the 419 and 433 Da linkers, respectively. (C and D) were obtained from the peptide SHAYNSAPVLYLGFDVAN of the CTD protein A0A1P8JBQ3, which were modified with the 419 and 433 Da linkers, respectively. The 419 Da linker consists of 88, 146 and 203 Da, and the 433 linker consists of 88, 160 and 203 Da components. The peak corresponding to the peptide mass (highlighted in yellow) is only present in (A). All labelled ions are doubly charged.

To further confirm the glycan sequences, additional CID scans were performed on the glycopeptide precursors that exhibited the 204 *m/z* ion. MS2-level CID spectra for two different C-terminal peptides are shown, both modified by the 419 and 433 Da linking glycans (figure 2). The modification components were confirmed to be (88+146+203) and (88+160+203) Da, respectively.

### 3.4. Identification of cleavage sites from released CTDs

Proteins released into the culture fluid during *P. intermedia* growth were separated by SDS-PAGE and the region of the gel below the 10 kDa marker was sliced into four segments for in-gel digestion and LC-MS/MS analysis. The data were then searched for CTD peptides, particularly those that indicated the sortase cleavage sites. CTD peptides from a total of 12 cargo proteins were identified (table 2). Of these, three exhibited non-tryptic cleavages on the N-terminal side, consistent with sortase cleavage, and one protein, A0A1P8JCJ7 produced a tryptic peptide that coincided with the predicted cleavage (table 2). Of note, the CTDs of all 12 cargo proteins were of the ‘long’ variety. Potentially, the short CTDs were too small to be resolved.

**Table 2.** MS identification of peptides within cleaved CTDs.

accession	#CTD Pep.^a^	putative sortase-cleaved peptide sequence^b^	Mascot score	E-value
A0A1P8JBQ3	5	N/NIGNAHVAGETFFYDANSHMMTFGK	81.3	2.9E−08
A0A1P8JEQ6	3	T/SLEMIEQDVHNVSISVYGSVLR^c^	80.7	2.7E−08
A0A1P8JCQ5	3	T/GINSAETPNKTETPYIVDK	53.6	9.4E−06
A0A1P8JCJ7	2	K/GVTTASADLTVVR^d^	34.0	0.00075
A0A1P8JCV6	3	not found		
A0A1P8JFN1	3	not found		
A0A1P8JC44	2	not found		
A0A1P8JDG2	2	not found		
A0A1P8JE01	2	not found		
A0A1P8JEV8	2	not found		
A0A1P8JG17	2	not found		
A0A1P8JCT5	1	not found		

^a^ Usually the CTD can be cleaved by trypsin into several peptides, only one of which (the N-terminal peptide) provides the sortase cleavage site. This column provides the number of unique peptide sequences identified.

^b^ The sequence of the most N-terminal peptide identified that could be consistent with sortase-cleavage of the CTD. Usually this cleavage is non-tryptic.

^c^ Many other cleavages were also observed for this peptide, suggesting a ‘ragged’ N-terminus. The cleavage shown is the most N-terminal of those observed. It is uncertain whether the other cleavages are by the sortase or if this CTD is prone to degradation by aminopeptidases.

^d^ This predicted cleavage is also a tryptic cleavage site and therefore may have been cleaved by trypsin.

### 3.5. Sequence analysis of the CTDs

**Figure 3.**
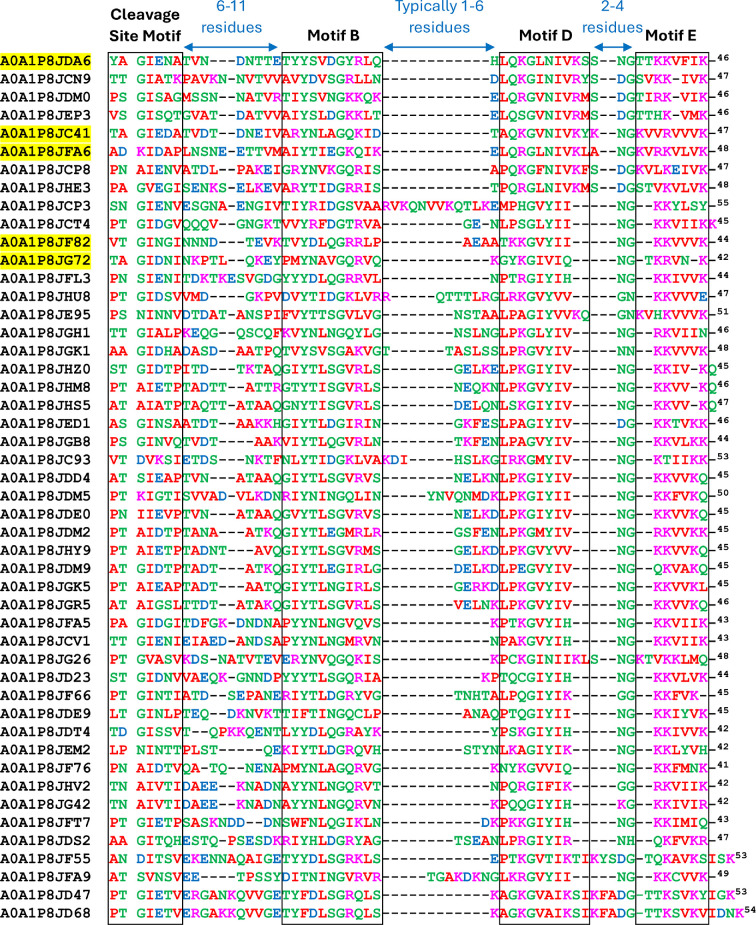
Multiple sequence alignment of ‘short’ CTDs and their cleavage sites. The 48 predicted short CTDs were aligned by Clustal Omega with subsequent manual adjustment. Cargo with experimentally identified cleavage sites are highlighted in yellow, with the remainder being predictions only. The cleavage site is indicated by the gap between positions 2 and 3 in the alignment while the approximate boundaries of the cleavage site motif, and conserved motifs B, D and E are indicated by the boxes. The Uniprot accession numbers for each protein are listed before each sequence. The numbers on the right represent the number of amino acid residues from the cleavage sites. Amino acids with similar biochemical properties are shown in the same colour. The boundaries of motifs B, D and E were defined according to their homology to the previously proposed CTD motifs in *P. gingivalis* and *T. forsythia* [28].

**Figure 4.**
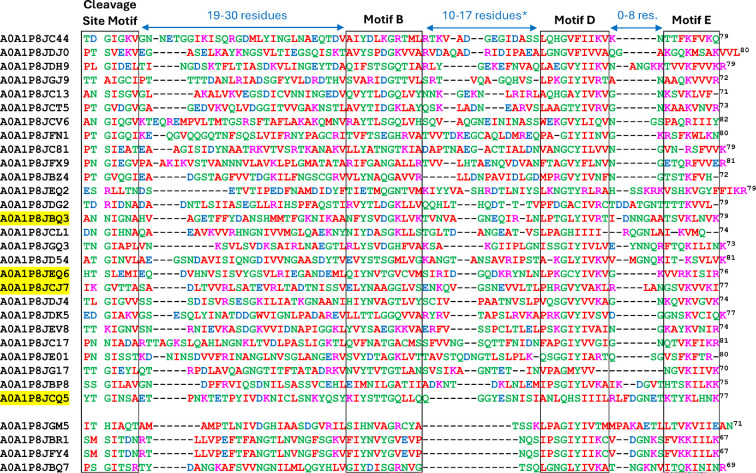
Multiple sequence alignment of ‘long’ CTDs and their cleavage sites. The 32 predicted long CTDs were aligned by Clustal Omega with subsequent manual adjustment. Cargo with experimentally identified cleavage sites are highlighted in yellow, with the remainder being predictions only. The cleavage site is indicated by the gap between positions 2 and 3 in the alignment while the approximate boundaries of the cleavage site motif, and conserved motifs B, D and E are indicated by the boxes. The Uniprot accession numbers for each protein are listed before each sequence. The numbers on the right represent the number of amino acid residues from the cleavage sites. Amino acids with similar biochemical properties are shown in the same colour. Since the alignment between the cleavage site motif and Motif B was very poor and full of gaps, this region was unaligned with all gaps placed together. The last four sequences are of medium length and have five predicted β-strands while the longer CTDs have seven predicted β-strands. *The gap length between motifs B and D excludes the final four sequences.

The sequences of the 48 short and 32 long CTDs were aligned separately (figures 3 and 4) with manual adjustments especially in the cleavage region as informed by the cleavage site data. In total, nine cleavage sites were determined experimentally (tables 1 and 2), and the accession numbers of these are highlighted yellow. The difference in length between the two groups was mainly accounted for in the distance between motifs. Long CTDs utilized 19−30 aa between the cleavage site motif and Motif B compared to 6−11 aa in the short CTDs. Similarly, between Motifs B and D, the distance was 10−17 aa for long CTDS, and only 1−6 aa for short CTDs (figures 3 and 4). A sub-group of four sequences at the bottom of figure 4 exhibited short distances between Motifs B and D (figure 4).

**Figure 5.**
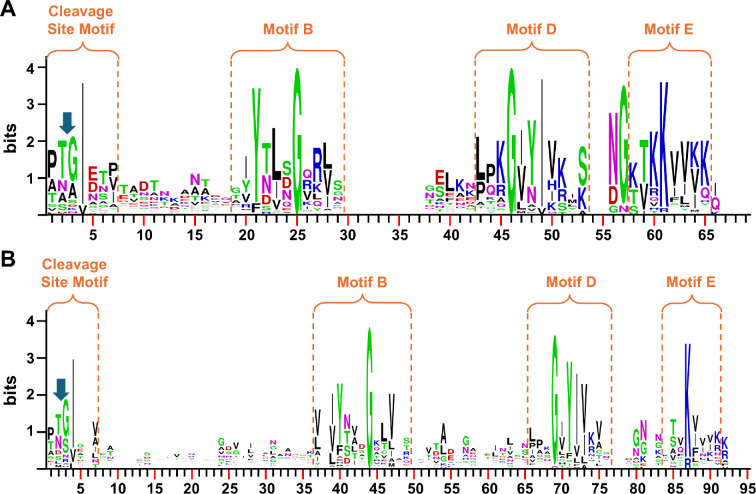
Sequence logo representations of the conserved motifs in CTDs. Amino acid logos were produced by WebLogo using the multiple sequence alignments shown in figures 3 and 4. (A) Short CTDs. (B) Long CTDs. The boundaries of motifs B, D and E were defined according to their homology to the previously proposed CTD motifs in *P. gingivalis* and *T. forsythia* [28]. A larger letter indicates a higher level of conservation of that particular amino acid at that position. Amino acids with similar biochemical properties are colour-coded using the same colour: blue, basic amino acids (Arg, His, Lys); red, acidic amino acids (Asp, Glu); green, polar amino acids (Ser, Thr, Cys, Tyr, Gly); black, hydrophobic amino acids (Ala, Val, Leu, Ile, Met, Phe, Trp, Pro); purple, Asn and Gln.

To facilitate the analysis of conservation patterns within the aligned sequences, logo representations were generated (figure 5). The larger size of a letter within the logo indicates a higher frequency and thereby a higher level of conservation of that amino acid at that specific position. Four motifs were apparent in the logos, namely the cleavage site motif, and Motifs B, D and E, and in general these reflect the published motifs from other species [28]. CTD cleavage in both groups was preferred at the consensus sequence PT/GI, within which Ile was the most conserved residue. In both, the Gly residues were absolutely conserved in Motifs B and D (figure 5). In Motif B, the Tyr was very strongly conserved in both, while specific to the short CTDs, a basic residue (Arg or Lys) was preferred at position 27 (figure 5A). In Motif D, the usual GxY motif was strongly conserved in both groups, but in the short CTDs, a sub-group of sequences (the top 8 in figure 3), utilized GxN instead. C-terminal to the Y or N was a highly conserved Ile in both groups. Motif E was similar in both groups, characterized by an absolutely conserved basic amino acid (Lys or Arg); however, the short CTDs preferred to have an extra basic residue at position 60 (figure 5A).

### 3.6. *In silico* structural analysis of the CTDs

**Figure 6.**
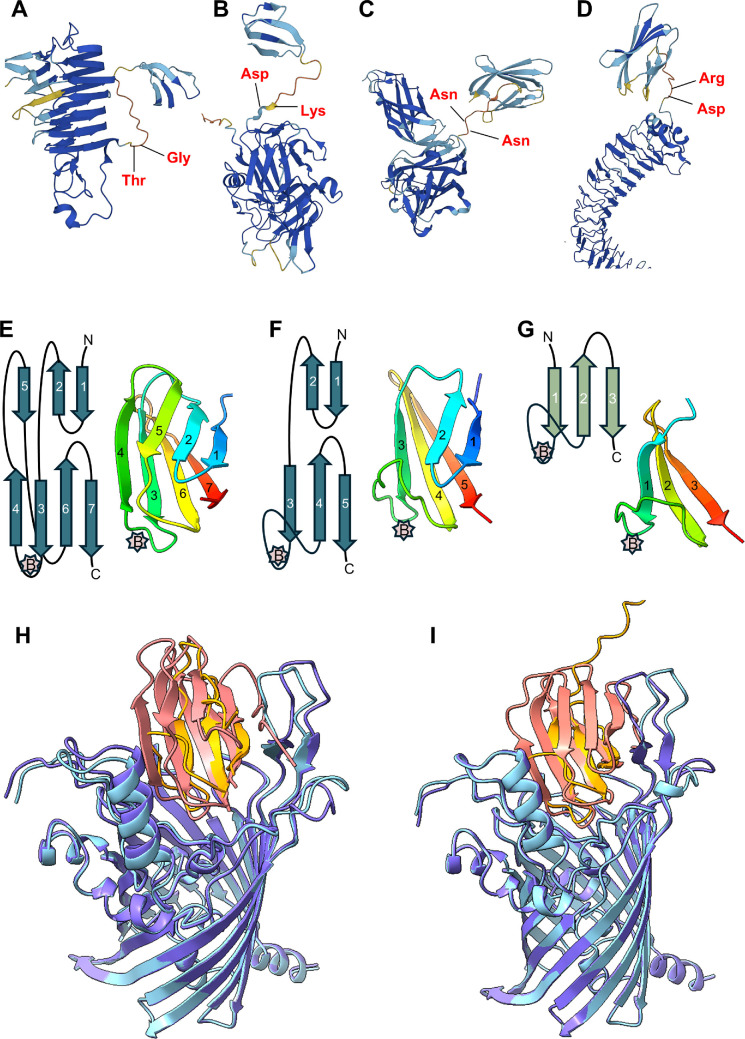
Structural predictions of short and long CTDs. (A–D) AlphaFold models of four cargo proteins, (A) A0A1P8JCN9, (B) A0A1P8JFA6, (C) A0A1P8JBQ3 and (D) A0A1P8JDG2, showing the cleavage sites of two short CTDs (A,B) and two long CTDs (C,D). The cleavage sites shown in (B) and (C) were determined experimentally, while those shown in A and D are predictions only. (E–G) AlphaFold model of a representative (E) long CTD (A0A1P8JDG2), (F) medium CTD (A0A1P8JBR1) and (G) short CTD (A0A1P8JFA6) along with their respective topological diagrams. The approximate location of Motif B is shown by the pink stars. (H–I) AlphaFold models of short/long CTD-PorV complexes. (H) Overlay of A0A1P8JDA6-PorV with A0A1P8JBQ3-PorV complexes. (I) Overlay of A0A1P8JCN9-PorV with A0A1P8JCJ7-PorV complexes.

To validate the cleavage site predictions (figures 3 and 4), AlphaFold models of each cargo protein were manually inspected to determine the location of these sites within each predicted structure. Figure 6A–D provides the structural evidence of four proteins, including two CTD proteins with identified CTD cleavage sites. In each case, the predicted or identified cleavage site was located in the flexible linker joining the folded portion of the CTD with the folded N-terminal domain, even though, three of the chosen sites were non-typical, being N-terminal to Lys (figure 6B), Asn (figure 6C) and Arg (figure 6D) instead of the preferred small residues, Gly, Ala and Ser [28].

The structural basis for the difference between short and long CTDs was also investigated by AlphaFold3 modelling. The long CTDs were predicted to be a β-sandwich of four β-strands packed against three β-strands (figure 6E) with the exception of the four sequences (at bottom of figure 4) which exhibited a β-sandwich of three β-strands packed against two β-strands (figure 6F). The short CTDs were typically predicted to have just one 3-stranded β-sheet (figure 6G). In some short CTDs, the ‘loop’ between strands 1 and 2 was predicted to include a short β-strand or α-helix. Since CTDs are predicted and known to bind to PorV [21,22,47] in other species, we also modelled the binding of short and long CTDs to *P. intermedia* PorV. Two pairs of short and long CTDs were predicted to bind to PorV in the same manner with strands 1, 2 and 3 of short CTDs being structurally equivalent to strands 3, 6 and 7 of long CTDs (figure 6H–I).

## 4. Discussion

A hallmark of the T9SS is its attachment system that utilizes the novel Gram-negative sortase, PorU to cleave the CTD signal of cargo and simultaneously conjugate them to a cell-surface polysaccharide such as LPS [12,36]. For delivery to the attachment complex, cargo must first be secreted through the translocon and bind to PorV via their CTDs [21]. In our previous study, we showed that *P. intermedia* was unusual in producing T9SS cargo with short CTDs of less than 60 aa amino acids [28], and therefore, we endeavoured to study the cargo from this organism with respect to their modification, CTD cleavage and predicted structural characteristics. Of note, all of the 80 predicted cargo were shown to be expressed in our recent glycoproteome study [34].

Using HMM, we predicted 48 ‘short’ and 32 ‘long’ CTDs. The length of the predicted short CTDs ranged from 43 to 55 aa residues, with the cleavage site confirmed experimentally for five sequences (figure 3). The motifs in both short and long CTDs are similar to those in other species; indeed, our previous HMM study utilizing *P. gingivalis* and *T. forsythia* CTDs as the input was capable of predicting most of them [28]. The unique feature of the short CTDs is their novel predicted structure employing just three β-strands as compared to the long CTDs with seven β-strands (figure 6). The predicted structures of the long CTDs are consistent with the published structures and models of several CTDs from *P. gingivalis* T9SS cargo revealing a common β-sandwich fold comprising four β-strands packed against three β-strands [25,26,48,49]. Furthermore, AlphaFold modelling of the RgpB-PorV shuttle complex [47] showed that PorV principally interacts with the major motifs (B, D and E), which are located at the same end of the 4-stranded β-sheet, with the 3-stranded β-sheet sitting on top where it plays a minimal role in the interaction. The loop between strands 3 and 4 contains Motif B and penetrates deepest into the PorV barrel. The short CTDs of *P. intermedia* appear to minimalize the structural requirement for PorV-binding, eliminating the top β-sheet and reducing the bottom sheet to just three full β-strands (figure 6G). Importantly, the locations of Motifs B, D and E are conserved, and remarkably, AlphaFold3 modelling of PorV shuttle complexes suggest that short CTDs bind to PorV in exactly the same way as the long CTDs (figure 6H–I). Interestingly, intermediate length CTDs were predicted to form a 5-stranded β-sandwich (figure 6F), similar to the published structure of the Hbp35 CTD of *P. gingivalis* [50]. Mutagenesis studies in *P. gingivalis* demonstrated that only the C-terminal 22 residues of Hbp35 encompassing the last two β-strands (containing Motifs D and E) were absolutely required for the secretion and attachment of GFP [51]; however, mutagenesis of Motif B within the RgpB cargo prevented its secretion [48]. The short CTDs of *P. intermedia* support the requirement for the conserved positioning of a loop containing Motif B together with the final two β-strands.

The PorV-cargo models also help explain that CTD cleavage is analogous in both short and long CTDs as the cleavage sites are consistently located within the coiled region between a structured domain of the mature protein and the structured portion of the CTD. Interestingly, both long and short *P. intermedia* CTDs have a strongly conserved isoleucine residue at the + 2 position relative to the cleavage site that is not evident in other species [28]. In contrast, the small residue (G, S, A) usually favoured in the + 1 position appears less crucial in *P. intermedia* with R, K, H, D and N also shown or predicted to be tolerated (figures 3–5).

**Figure 7.**
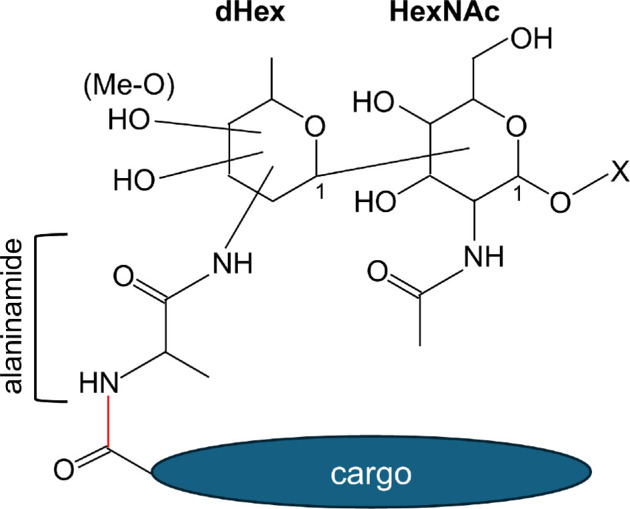
Structure of the C-terminal linking sugars. After cleavage of the CTD, the new C-terminus of the cargo is covalently bonded to the linking sugars via alanine. The new amide bond is shown in red. The alanine is N-linked to a dHex sugar at an unknown linkage position. In the Δmass 419 linker, two OH groups are also present at unknown positions, while in the Δmass 433 linker, one of these is methylated (Me-O). The dHex sugar is presumably linked to the adjacent HexNAc residue through the C1 position. Prior to deglycosylation, the HexNAc is expected to be linked to a large polysaccharide such as LPS—marked with an X.

We also showed that cargo with both short and long CTDs can be glycosylated at their matured C-terminus. Our earlier study indicated that the cargo proteins of *P. intermedia* were extensively glycosylated over a broad molecular weight range consistent with conjugation to LPS [28], hence the need to use a deglycosylation procedure to simplify the identification of glycosylated peptides, as previously achieved for *P. gingivalis* and *T. forsythia* cargo [24,36]. The linking sugar was determined to be an N-alanyl deoxyhexose that was further linked to HexNAc. These two sugars alone were left linked to the proteins after our TFMS deglycosylation procedure. Although the data were searched extensively, no further sugars could be identified. We have shown that TFMS cleavage strongly favours the cleavage of dHex residues, suggesting that the third sugar may be dHex [34,38,39,52]. We also observed that TFMS causes artefactual arylation at the reducing end of cleaved sugars, but not to 2-N-acetyl sugars [36,53,54]. Taking this knowledge into account explains why the HexNAc residue was not arylated. The N-alanyl deoxyhexose was partially methylated. The tendency for both forms (± methyl) to lose water or methanol, respectively, suggests that methylation occurs on the most labile oxygen. The structure of the linking sugars and their connection to the cargo is shown in figure 7.

Previously, the linking sugars were deduced to be 2-N-seryl, 3-N-acetylglucuronamide in *P. gingivalis* and 2-N-glycyl, 3-N-acetylmannuronic acid in *T. forsythia*. These two sugars were shown to be the product of a novel biosynthetic pathway, designated as the Wbp/Vim pathway [36]. The Wbp portion of the pathway synthesizes a 2,3-di-N-acetylated glucuronic acid. The Vim enzymes subsequently switch out the acetyl group in the 2′ position replacing it with serine or glycine. The linking sugar in *P. intermedia* is substantially different, based on a simple dHex sugar with just the one substitution (N-alanyl) (figure 7). Consistent with this, *P. intermedia* lacks four of the key enzymes in this pathway, namely WbpB, WbpD, VimA and VimE. The most common dHex sugars are fucose, rhamnose and quinovose. N-acetylated forms of dHex sugars linked at various positions (2’, 3’, 4’) have been reported in the *O*-polysaccharide portions of LPS from other species [55–57]; therefore, it is not possible to speculate the position of the N-linkage. N-linked alanine has also been found in *O*-polysaccharides, for example in *Escherichia coli O167* and *E. coli O123* [57]; however, to the best of our knowledge, the biosynthesis pathway that explains the transfer of alanine to a dHex residue is unknown. In any case, it is noteworthy that although the PorU sortase and CTD cleavage are conserved between *P. intermedia* and other T9SS species, the structure and biosynthesis of the linking sugar is not. Our finding opens up the possibility that several different biosynthetic pathways may exist across T9SS species within the *Bacteroidota* phylum to enable a diverse suite of linking sugars to be utilized.

In summary, this study builds upon our recent report that initially unveiled the *O*-glycosylation system in *P. intermedia* [34], by further characterizing another glycosylation system—C-terminal-modification of T9SS cargo proteins. We identified N-alanyl-dHex-HexNAc and N-alanyl-(Me-dHex)-HexNAc as the linking sugars in LPS that anchor CTD proteins to the cell surface. Additionally, through multiple alignments of CTD protein sequences, we revealed that the CTDs have conserved cleavages sites and motifs as other *Bacteroidetes* species, yet *P. intermedia* is the only species within this phylum reported to possess both long and short CTDs.

## Data Availability

The mass spectrometry proteomics data have been deposited to the ProteomeXchange Consortium via the PRIDE partner repository with the dataset identifier PXD057272 and 10.6019/PXD057272. Supplementary material is available online [58].
